# A Systematic Review of Changes in Marine Mammal Health in North America, 1972-2012: The Need for a Novel Integrated Approach

**DOI:** 10.1371/journal.pone.0142105

**Published:** 2015-11-18

**Authors:** Claire A. Simeone, Frances M. D. Gulland, Tenaya Norris, Teresa K. Rowles

**Affiliations:** 1 The Marine Mammal Center, Sausalito, California, United States of America; 2 Office of Protected Resources, National Marine Fisheries Service, National Oceanic and Atmospheric Administration, Silver Spring, Maryland, United States of America; Sonoma State University, UNITED STATES

## Abstract

Marine mammals are often cited as “sentinels of ocean health” yet accessible, synthesized data on their health changes that could effectively warn of ocean health changes are rare. The objectives of this study were to 1) perform a systematic review of published cases of marine mammal disease to determine spatial and temporal trends in disease from 1972–2012, including changes in regions and taxa affected and specific causes; and 2) compare numbers of published cases of neoplasia with known, hospital-based neoplasia records to explore the causes of discrepancy between numbers of published cases and true disease trends. Peer-reviewed literature was compiled, and data were collected from The Marine Mammal Center database in Sausalito, California for comparison of numbers of neoplasia cases. Toxicoses from harmful algal blooms appear to be increasing. Viral epidemics are most common along the Atlantic U.S. coastline, while bacterial epidemics, especially leptospirosis, are most common along the Pacific coast. Certain protozoal and fungal zoonoses appear to be emerging, such as *Toxoplasma gondii* in southern sea otters in California, and *Cryptococcus gattii* in cetaceans in the Pacific Northwest. Disease reports were most common from California where pinniped populations are large, but increased effort also occurs. Anthropogenic trauma remains a large threat to marine mammal health, through direct mortality and indirect chronic disease. Neoplasia cases were under-reported from 2003–2012 when compared to true number of cases, and over-reported in several years due to case duplication. Peer-reviewed literature greatly underestimates the true magnitude of disease in marine mammals as it focuses on novel findings, fails to reflect etiology of multifactorial diseases, rarely reports prevalence rather than simple numbers of cases, and is typically presented years after a disease first occurs. Thus literature cannot guide management actions adequately, nor inform indices of ocean health. A real-time, nationally centralized system for reporting marine mammal disease data is needed to be able to understand how marine mammal diseases are changing with ecosystem changes, and before these animals can truly be considered ‘sentinels of ocean health’.

## Introduction

In recent decades, the altered state of marine ecosystems around the world have been increasingly linked to anthropogenic activities, such as urbanization, pollution, and climate change [1][2]. This has led to greater awareness of the importance of ecosystem health among managers, policy makers, and the general public. Although there are numerous ways to assess the health of an ecosystem [3], two general approaches are used most frequently. One approach initially reported by Hall and Kerr [4] is to measure multiple environmental parameters to develop an integrated assessment of health. This has been used most recently by Halpern (2012) [5] to establish an Ocean Health Index, which uses ten widely held goals to quantify ocean health. The other common method for assessing ecosystem health characterizes health changes in indicator species. These sentinels are usually invertebrates, but may include large mammals that belong to upper trophic levels and attract public support for conservation [6][7].

Marine mammals face numerous threats to health from both anthropogenic and natural stressors. Recognition of the impacts of harvest and incidental takes in fisheries on marine mammal populations led to passage of the Marine Mammal Protection Act (MMPA) in the United States in 1972 to protect these animals and the ecosystems upon which they depend [8]. Since passage of the MMPA, large-scale marine mammal die-offs and uncertainty as to their causes led to additional legislation in 1992 (Title IV of the MMPA) that called for establishment of the Marine Mammal Health and Stranding Response Program (MMHSRP). Title IV tasked the MMHSRP to oversee the collection and dissemination of information on the health of marine mammal populations; assessment of these health trends associated with biological, chemical, and physical environmental parameters; and development of an effective and efficient response to marine mammal unusual mortality events [9]. The overarching goals of these legislative actions were to better understand and reduce threats to marine mammals, and to increase response to unusual mortality events. Since enactment of the MMPA, some marine mammal populations have recovered, such as the northern elephant seal (*Mirounga angustirostris*) and the eastern North Pacific gray whale (*Eschrichtius robustus*) [10][11]. However, anthropogenic impacts, including fisheries interactions [12], ocean noise [13], ship strikes [14], and marine debris [15], continue to threaten marine mammal population recovery. Additional threats to marine mammal populations have been realized, and include exposure to pollutants, infectious diseases, and harmful algal blooms [16] as well as sea ice recession and rising sea temperatures [17].

Amongst marine organisms, marine mammals often are cited as sentinels for ocean and human health because they are long-lived, feed at upper trophic levels, have fat stores that accumulate anthropogenic toxins, and are vulnerable to many of the same pathogens, toxins, and chemicals as humans [18][19][20][21]. Marine mammals are charismatic marine species, and as such, mortalities and changes in their health draw attention from the public, regulators, and managers to issues such as ocean health, ocean pollution, pathogen spread, and harmful algal bloom effects. Publications on marine mammal diseases are increasing, but whether this trend is reflective of true spatiotemporal trends, as is apparent in other marine organisms such as corals, birds, and fishes, is unclear [22][23][24]. In addition, it has been recognized that lack of a shared definition of ecosystem health which includes health metrics from individual species may prevent scientists and managers from properly assessing risks to protected species and from making informed management actions [25].

Efforts have been underway to better characterize and understand marine mammal health. Health has been defined not simply as a lack of disease, but as complex interactions between biological, social, and environmental parameters that affect an organism’s capacity to cope with ecosystem changes, and a population’s resilience [25]. Thus, scientists have attempted to characterize marine mammal health using both evidence of disease and response to external stressors [26][27]. Whereas considerable research has been performed to assess the environmental stressors associated with changes in marine mammal population size, behavior, and habitat use [19][28][29], assessing trends in marine mammal diseases, beyond reports on single species and over small geographic areas, has proven difficult. In the United States, this is in part because no centralized database exists for marine mammal disease data.

Other researchers have investigated disease trends by analyzing published reports as a proxy for understanding whether disease outbreaks are increasing [22][24][30][31][32]. While peer-reviewed publications are typically not intended to provide the comprehensive data required to track disease trends in populations through time, they are currently the only source for which this can be attempted for marine mammals in the United States. For this reason, here we analyze 40 years of published reports on marine mammal disease in North America to fulfill two objectives. The first is to explore etiology of disease and affected taxa between 1972 and 2012, and to evaluate apparent spatial and temporal trends in marine mammal disease reports during this period. The second objective is to explore differences in numbers of disease cases calculated from published reports from numbers of known cases, to investigate biases in reporting.

## Materials and Methods

### Literature sources

**Fig 1:**
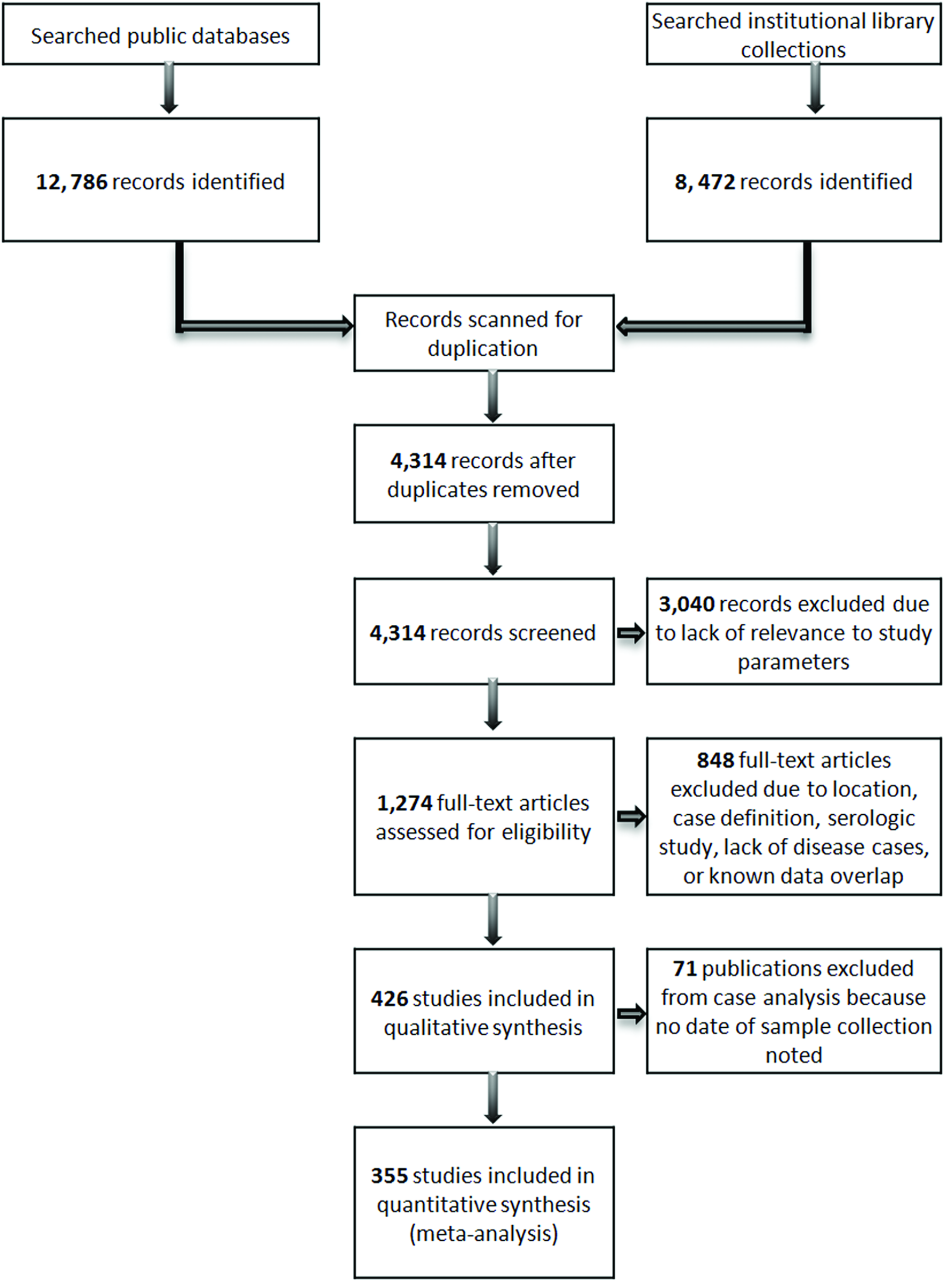
Process for selection of publications included in the systematic review.

Peer-reviewed journal articles, abstracts and reports from conferences, symposia, and workshops, and U.S. government technical memoranda published between 1972 and 2014 and printed in English that documented cases of marine mammal diseases were compiled (Fig 1). Multiple public databases were searched, including Google Scholar, PubMed, Medline, ISI Web of Knowledge, and Scopus. Several institution library collections were used to gain access to publications not readily available online, including those at the National Marine Mammal Foundation, The Marine Mammal Center, the Marine Mammal Commission, and National Marine Fisheries Service regional Science Centers. Searches focused on terms related to marine mammal health and disease (i.e. illness*, *mortality*, lesion*, disease*, patho*) as well as etiologic categories (i.e. infec*, toxi*, bacter*, myco*, parasite*, protozoa*, *virus*, *viral*, neoplas*, trauma*). Terms were initially searched in association with target marine mammal species with known geographic ranges in North America (i.e. ‘northern fur seal’ AND ‘disease*’). Reports of wild marine mammals from the United States and Canada were included in this study. All species of marine mammals were included (pinnipeds, cetaceans, sirenians, sea otters, walruses, and polar bears). Cases were characterized by taxa but not species, because many reports included multiple species and did not differentiate between species for each case. Reports on marine mammals in captive facilities were excluded, due to the possibility of animal movements between regions, and potential differences in exposure to environmental health risks that may not be similar to wild populations.

“Publication” refers to a single report regardless of the type (e.g., abstract or peer-reviewed journal article) and number of individual animals included. Each publication was counted even when it was explicitly stated that one or more other publication also reported on the same animal(s) or event(s). However, cases that were reported in multiple publications were counted only once in the analysis. Publications were categorized by year of publication. All publications included in either the final qualitative or quantitative syntheses are listed in S1 Appendix.

### Calculated case designation

Within each publication, a “case” refers to each documented account of a disease or health finding in an individual animal. A single animal with multiple disease processes would be assigned to more than one category and counted multiple times as distinct cases. The total number of cases reported is therefore a calculation (“calculated cases”), and not reflective of the actual number of individual animals. This method was chosen to more accurately assess each health category, without giving the false impression of higher numbers of total animals. If multiple publications described overlap in reporting on the same cases, the cases were counted only once in the analysis. Cases were included, however, when there was potential overlap but no explicit statement of duplicate reporting. Similarly, if a conference proceedings and a peer-reviewed manuscript by the same authors described the same cases or event, the cases reported in the conference proceedings were removed from the analysis. If a publication did not report the year(s) in which the study was conducted, it was included in analysis of publications, but data from it were not included in analysis of cases.

#### Date

Calculated cases were assigned to a year by date the sampling was performed. When cases were collected over a range of years, with no designation to the exact year from which each case originated, the cases were divided equally over the years within the reported date range. Calculated case years ranged from 1972–2012; thus, there was not complete overlap in dates of publications and cases.

#### Location

**Fig 2:**
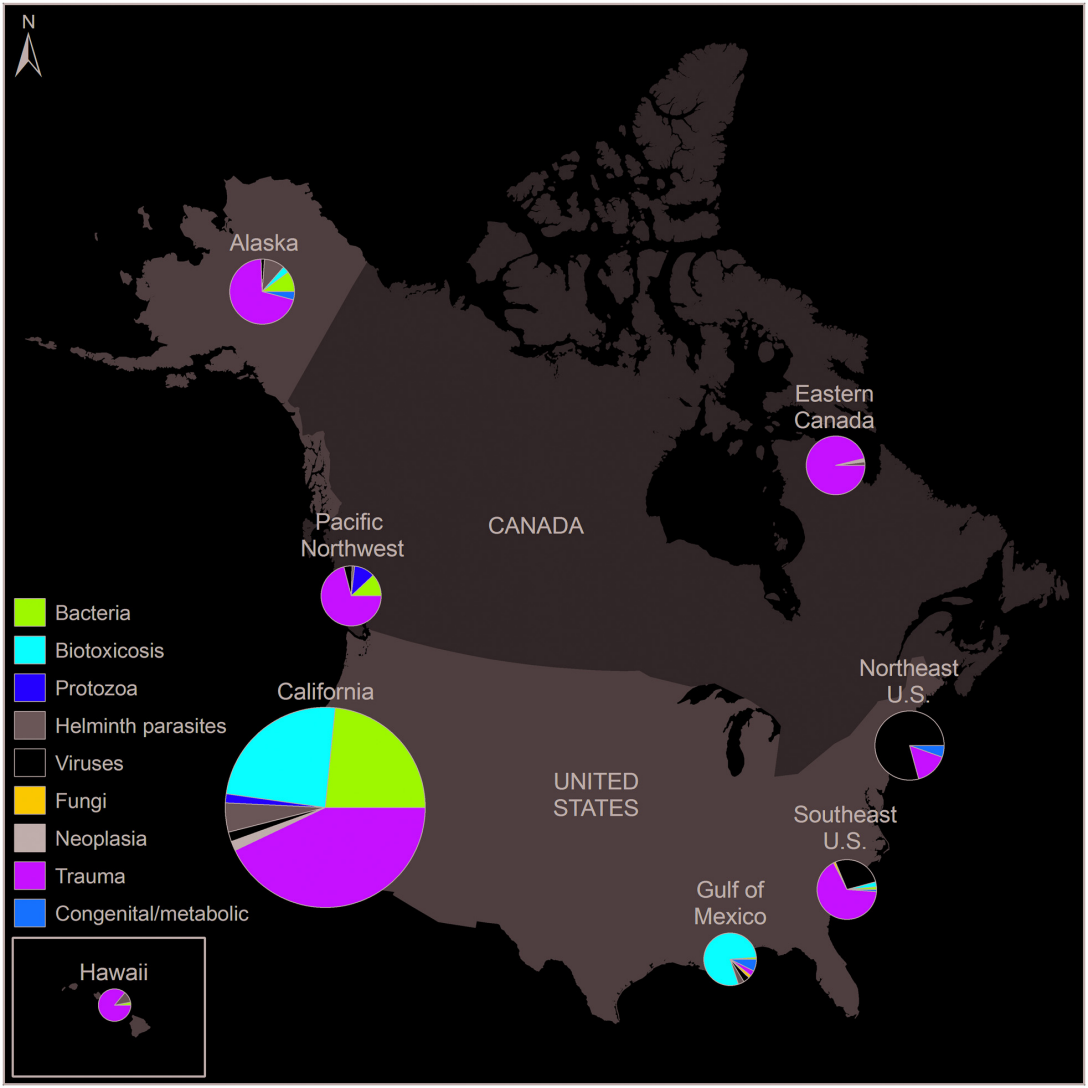
Proportion of calculated cases extracted from published reports on marine mammal disease for each disease category and eight coastal regions of the United States (U.S.) and Canada. Symbol sizes represent the cumulative number of calculated cases per region that were associated with clinical disease, which ranged from 425 calculated cases for Hawaii to 16,275 calculated cases for California. Map created using ArcMap 9.3 (ESRI, Redlands, CA).

Based on the location of animal sampling, calculated cases were allocated to one of eight coastal regions in North America (Fig 2): Alaska (including several reports from Yukon Territory), Pacific Northwest (Oregon [OR], Washington [WA], and British Columbia [B.C.]), California, Northeast U.S. (extending from Maine south to Maryland), Southeast U.S. (extending from Virginia south to the Atlantic coast of Florida), Gulf of Mexico (Gulf coast of Florida extending west to Texas), Eastern Canada (any coastal area east of Nunavut and Manitoba), and Hawaii. These regions reflect the spatial groupings commonly encountered in disease reports (e.g., cases from WA, OR, and B.C. typically were grouped together in a single publication, and cases sampled from VA to FL were grouped in several publications), although this may not reflect regional movement of marine mammal species, or regions for management activities. When cases from a single publication were collected from more than one region, with no designation to the exact number of cases from each region, the cases were divided equally among regions.

#### Disease category

Calculated cases were categorized by disease agent. Ten disease categories were used: infectious disease, which was sub-divided into viruses, bacteria, fungi, helminthes, and protozoa; non-infectious disease, which was sub-divided into neoplasia, biotoxicosis, and congenital/metabolic; and trauma, which was sub-divided into anthropogenic (such as entanglement or ship strike) and non-anthropogenic/unknown source (such as shark bite). For infectious diseases, only cases that documented a pathogen, via histology, isolation, culture, or PCR, were included. Serologic data were excluded as they report only response to pathogen exposure but not evidence of active infection, and only a few serological tests have been validated for marine mammals. Notably, marine mammals that were fisheries by-catch were excluded from the dataset because by-catch was considered accidental capture of an otherwise healthy animal. In addition, malnutrition was not included as a separate disease category because this health finding typically was reported along with another disease process. The majority of publications did not indicate when malnutrition was primary or secondary to a separate disease. Cases were also grouped by whether they described (1) clinical disease, or (2) the isolation of an organism or toxin without evidence of clinical disease. Clinical disease was defined as either pre-mortem clinical findings of disease or post-mortem gross or histologic evidence of response to a stressor.

### Comparison of number of cases in published reports with hospital-identified cases

To test the hypothesis that the published literature does not reflect true trends in marine mammal disease, calculated cases of neoplasia from the published literature were compared with the number of stranded California sea lions (*Zalophus californianus*) admitted to The Marine Mammal Center (TMMC; Sausalito, CA) with urogenital carcinoma between 1975 and 2012. The TMMC sea lion carcinoma dataset included animals that stranded in central and northern California (1,000 km of coastline from Mendocino to San Luis Obispo Counties), which represents most of the region in California with published cases of neoplasia. Gross necropsy and histopathology results from sea lions tagged in TMMC’s databases as carcinoma cases were reviewed to ensure historic cases were correctly classified. Published cases of neoplasia included California sea lions with urogenital carcinoma as well marine mammals from California and other regions with other neoplastic lesions (e.g., sarcoma and papilloma).

## Results

### Publications and overall calculated cases

**Fig 3:**
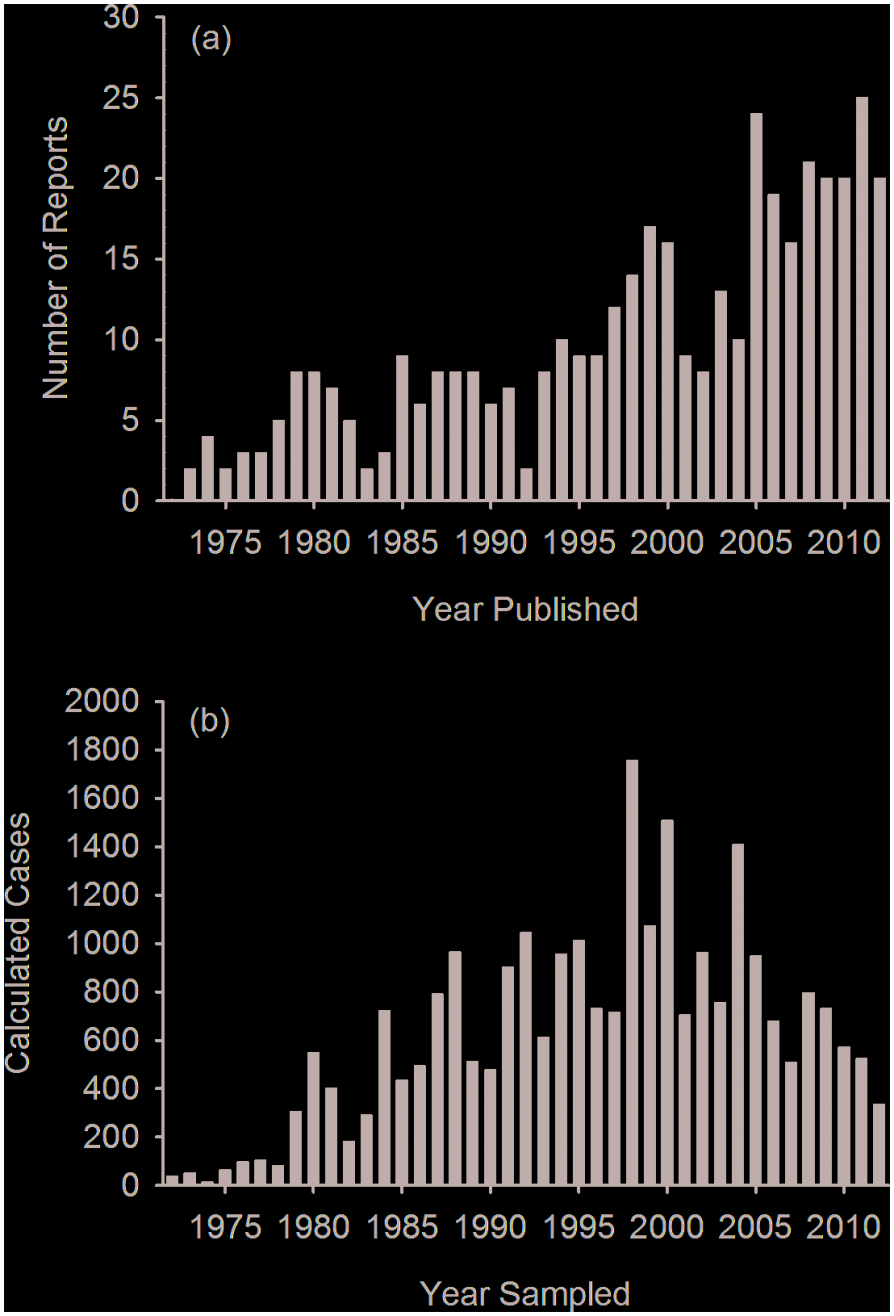
Annual number of (a) published reports on marine mammal disease and (b) calculated cases extracted from these reports for the United States and Canada, 1972–2012. When more than one disease process contributed to the cause of morbidity for an individual animal, it was assigned to more than one category and counted multiple times as distinct calculated cases.

A total of 426 publications met the criteria for inclusion in this study. The number of annual publications showed an increasing trend over time, with most of the increase occurring after 1996 (Fig 3a). Fourteen percent of the publications described disease findings in a single animal. Seventeen percent of publications did not document the year in which the study was conducted, which led to the exclusion of 71 publications from the calculated case analyses.

A total of 30,244 marine mammal calculated cases were documented in the publications. Annual number of calculated cases showed an increasing trend from 1972 through 1998, stayed relatively stable from 1998–2004, and then decreased until the end of the study period (Fig 3b). Of the total calculated cases, 85% (n = 25,820) were cases of clinical disease, rather than cases that described the presence of a disease-causing organism or biotoxin, without evidence of disease (Table 1). Sixty-three percent (n = 16,275) of all calculated cases reported were from California (Fig 2).

**Table 1:** Calculated cases divided into those describing clinical disease (“disease”) and those with only the disease-causing agent isolated (“isolation”) for the infectious disease and biotoxicosis categories, 1972–2012.

Health Category	Number Disease Cases	Number Isolation Cases	Proportion Disease	TOTAL
**Viruses**	2,306	447	0.84	2,753
**Fungi**	65	0	1.00	65
**Protozoa**	419	291	0.59	710
**Helminthes**	1,087	1,035	0.51	2,122
**Bacteria**	4,198	2,448	0.63	6,646
**Biotoxicoses**	4,950	203	0.96	5,153
**TOTAL**	**13,025**	**4,424**	**0.75**	**17, 449**

### Calculated cases by disease category

#### Bacteria

**Fig 4:**
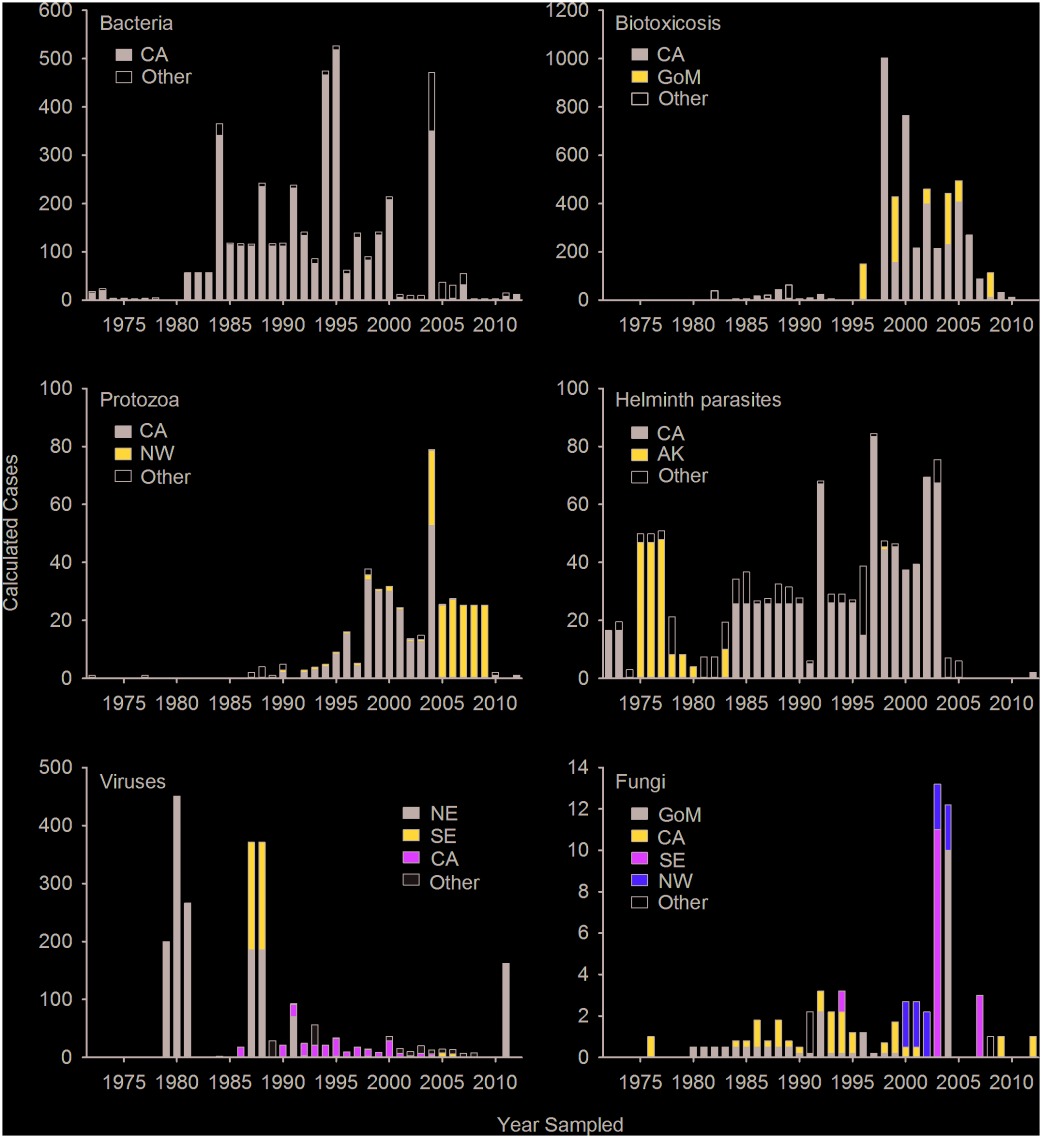
Annual number of calculated cases by disease category and region extracted from published reports on marine mammal disease. For each disease category, regions representing less than 10% of the cumulative number of calculated cases were grouped (other). CA = California, GoM = Gulf of Mexico, NW = Pacific Northwest, AK = Alaska, NE = Northeast U.S., and SE = Southeast U.S.

Bacterial cases represented approximately 20% of the total number of calculated cases reported (Table 1). Sixty-three percent (n = 4,198) of these cases were associated with clinical disease (Table 1). Cyclical epizootics associated with *Leptospira interrogans* serovar Pomona in California sea lions along the Pacific coast of Canada and the U.S. were the most frequent cause of disease attributed to a single bacterial species in marine mammals over the study period. The majority (90%) of these calculated cases were from California, with large peaks in 1984, 1994, 1995, and 2004 (Fig 4). No cases of leptospirosis were documented along the Atlantic coast or Gulf of Mexico.

Reports of bacterial isolation without indication of clinical disease (36% of total bacterial cases) typically characterized normal flora or the detection of antibiotic resistant strains. Isolated bacteria were occasionally speciated, but were most often grouped by genera and reported as mixed bacterial infections.

#### Biotoxicoses

Calculated cases in the biotoxicosis category comprised nearly 20% of the total number of cases. Reports of intoxication from harmful algal blooms were infrequent before 1998, and have since been recognized and reported as causing marked morbidity and mortality in marine mammals in California and the Gulf of Mexico (Fig 4). Most biotoxicosis calculated cases (95%) were associated with clinical disease (Table 1), and of those cases, 80% originated from California (Fig 2). Cases of biotoxicosis from California were almost exclusively reports of domoic acid affecting California sea lions. Eighteen percent of total biotoxicosis cases originated in the Gulf of Mexico, and biotoxicosis cases represented 79% of all cases in this region (Fig 2). Gulf of Mexico calculated cases of biotoxicosis were caused by brevetoxin or mixed brevetoxin, domoic acid, and okadaic acid toxicity. Biotoxicosis cases that reported detectable concentrations of toxin in tissues and/or bodily fluids, without associated clinical disease, were infrequently reported (4% of total biotoxicosis cases).

#### Protozoa

Nine calculated cases of protozoal disease were published prior to 1990, but over the last two decades, the number of protozoal disease cases appears to be increasing in marine mammals, primarily in benthic-feeding sea otters, found in U.S. and Canadian waters (Fig 4). The most commonly reported protozoal disease cases were in southern sea otters (*Enhdra lutris nereis*) and caused by *Sarcocystis neurona*, *Toxoplasma gondii*, or co-infection with these two parasites. Cases of these diseases were most common in southern sea otters in California from 1996–2004, with a 2004 mass mortality caused by *Sarcocystis neurona*, whereas cases in the Pacific Northwest were more recently documented (Fig 4). Regionally, the Pacific Northwest had the greatest proportion of protozoal disease cases (11%, Fig 2).

#### Helminth parasites

Fifty-one percent (n = 1,087) of calculated helminth cases were associated with clinical disease (Table 1). Annual calculated case numbers were steady through 2003, and originated primarily from California and Alaska (Figs 2 and 4). The last reported helminth disease case from Alaska, however, was in 1998, and since 2007 there have been few reported cases of helminth parasites associated with clinical disease (Fig 4). Parasites that caused significant morbidity and mortality included hookworms (*Uncinaria* sp.), causing anemia and peritonitis in northern fur seal (*Callorhinus ursinus*) and California sea lion pups; the lungworm *Otostrongylus circumlitis* causing disseminated intravascular coagulopathy in northern elephant seals (*Mirounga angustirostris*); thorny-headed worms (*Profilicollis* sp.) causing peritonitis in southern sea otters; and sinus flukes associated with neurologic disease in cetaceans.

Publications that reported the presence of helminth parasites, either as an incidental finding on necropsy, or isolated from feces (“isolation”), represented 49% of the calculated cases (Table 1). Helminth-isolation publications were common in the 1970’s, as species were being discovered in novel hosts. The focus shifted in the 1980’s to publications that included parasites as component of multiple findings associated with marine mammal disease.

#### Viruses

Viral epizootics occurred several times throughout the study period (Fig 4). These epizootics were regionally specific and occurred along the Atlantic coast of the U.S., but not the Pacific coast (Fig 2). There were 742 calculated cases of morbillivirus in bottlenose dolphins (*Tursiops truncatus*) along the Atlantic coast of the U.S. from 1987–1988. Morbillivirus outbreaks with fewer calculated cases occurred in bottlenose dolphins in the Gulf of Mexico in 1993–1994 (n = 34), and in harbor seals (*Phoca vitulina*) in the Northeast U.S. in 1992 (n = 68). Influenza also caused epizootics in harbor seals in the Northeast U.S., with reports of roughly 500 calculated cases in 1979–1981, and 162 calculated cases in 2011. In other regions, there were zero (Hawaii) to 250 (California) total viral calculated cases associated with clinical disease (Fig 2), a majority of which were herpesvirus cases.

#### Fungi

Fungal diseases were sparsely reported, and particular fungal species tended to be regionally specific (Figs 2 and 4). An increase in annual number of fungal calculated cases was noted in 2003–2004 (Fig 4). In 2003, the peak in dermal fungal disease cases was due to a single publication estimating prevalence of lacaziosis in bottlenose dolphins in Florida. In California, fungal cases were coccidioidomycosis reported in California sea lions (*Zalophus californianus*), southern sea otters, a northern elephant seal (*Mirounga angustirostris*), and a bottlenose dolphin. *Cryptococcus gattii* appears to be emerging in the Pacific Northwest, and in marine mammals there were reports in cetaceans starting in British Columbia and Washington in 2000, and later being reported in Hawaii (2008) and California (2009).

#### Comparison of numbers of published reports of neoplasia with numbers of hospital identified cases

**Fig 5:**
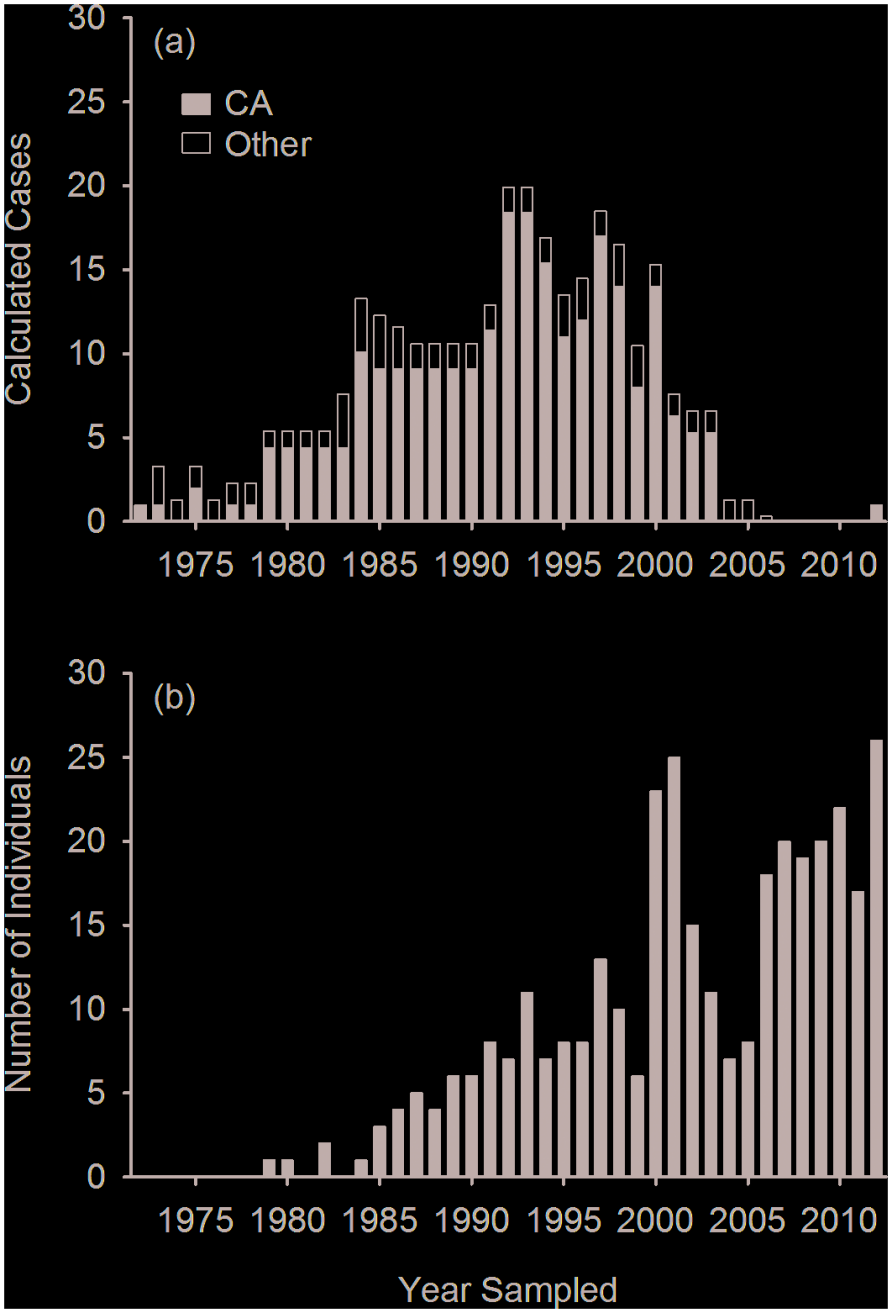
Annual number of (a) calculated cases of neoplasia extracted from published reports on marine mammal disease and (b) individual California sea lions (*Zalophus californianus*) with urogenital carcinoma extracted from The Marine Mammal Center dataset. All calculated cases for California from 1979–2003 were from California sea lions with urogenital carcinoma originating from TMMC’s dataset, except for one case per year in 1984, 2000, and 2001.

Neoplasia comprised 9% of publications (n = 38), and only 1% of calculated cases (n = 307). Reports of neoplasia in marine mammals were rare and sporadic in most regions (Fig 2), and 56% of reports (n = 23) described neoplasia in three or fewer individuals. Two populations were exceptions: California sea lions along the California coast, and beluga whales (*Delphinapterus leucas*) in the St. Lawrence Estuary, Canada. One to two calculated cases of mixed neoplasms were reported in the St. Lawrence Estuary beluga population from 1983–1999, but no cases have been reported in this population since 1999. There were four to 18 calculated cases of urogenital carcinoma reported in California sea lions annually from 1979–2003 (Fig 5a). Calculated cases of neoplasia peaked in the 1990’s, with only one reported case after 2006 (Fig 5a).

All calculated cases of neoplasia in California from 1979–2003 were from California sea lions admitted to The Marine Mammal Center (TMMC), with the exception of one case each in 1984, 2000, and 2001. No neoplasia cases were reported in California sea lions after 2003. Despite the absence of published reports of neoplasia in California sea lions from 2003–2012, cases of urogenital carcinoma in California sea lions examined at TMMC increased throughout the study period (Fig 5b). In addition, prior to 2000, the number of calculated cases of urogenital carcinoma in California sea lions from published reports originating from TMMC exceeded the number of individuals with this disease in TMMC’s dataset.

#### Trauma

**Fig 6:**
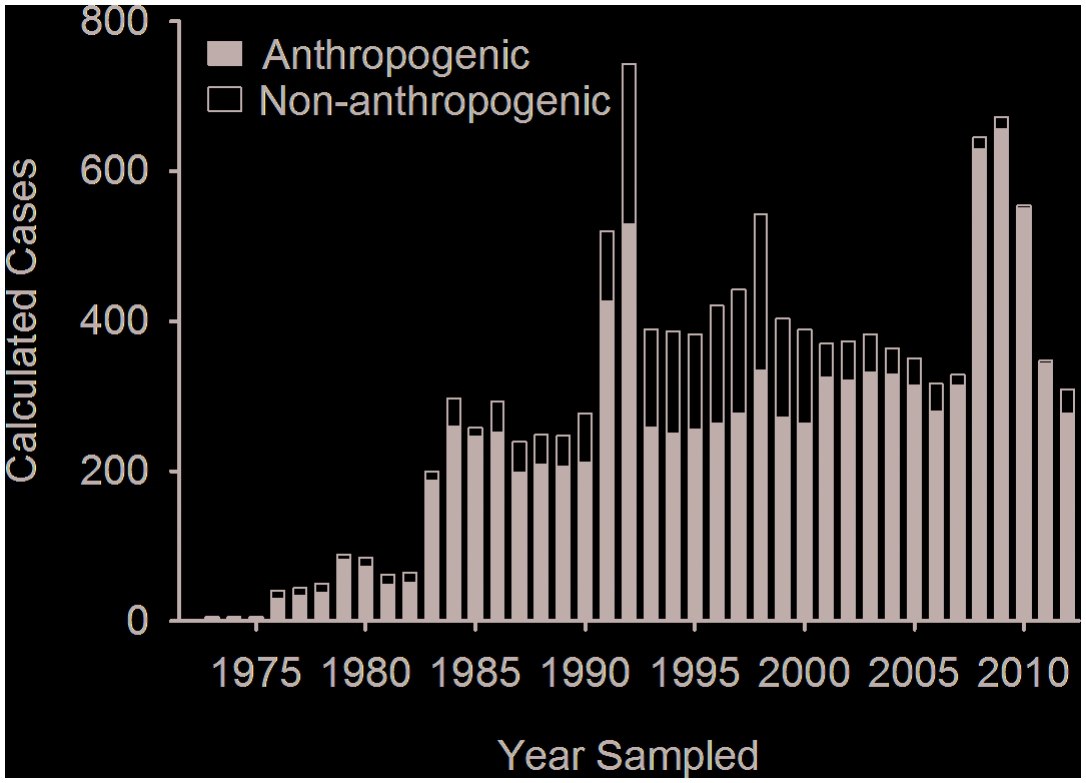
Annual number of calculated cases of trauma extracted from published reports on marine mammal disease. Source of trauma was designated as anthropogenic or non-anthropogenic/unknown.

Trauma cases were reported in greater numbers than any of the disease categories, representing 40% of total calculated cases, and were the most frequent category reported in six of the eight studied regions (Fig 2). The majority (82%) of calculated trauma cases were attributed to an anthropogenic source. Annual anthropogenic trauma cases were relatively stable, or increased slightly, throughout the study period with no recent decrease in calculated cases (Fig 6). Non-anthropogenic trauma calculated cases peaked in the 1990s, and decreased after this period (Fig 6). Publications documented issues common to marine mammals across regions: boat strikes of large whales, smaller cetaceans, and sirenians as well as entanglements of large whales and pinnipeds. Non-anthropogenic causes of morbidity were primarily predation, either by sharks on pinnipeds and sea otters, or by other marine mammal species or conspecifics.

#### Congenital/metabolic

Calculated cases of congenital or metabolic diseases were infrequently reported in marine mammals from the U.S. and Canada (Fig 2), with only 334 congenital or metabolic disease cases noted during the study period (1.1% of total cases). These cases often were reported as part of large-scale, multi-year studies that documented causes of illness and/or death in one or more species.

## Discussion

Numerous factors likely contributed to the increase in publications on marine mammal disease after 1996. Changes in surveillance techniques, such as the increase in the number of stranding response facilities in the United States, resulted in an increase in the number of carcasses for examination [22], following passage of Title IV of the MMPA in 1992 and establishment of the Prescott Marine Mammal Rescue Assistance Grant Program in 2000 [16]. Increased reporting of marine mammal disease events has also been attributed to an increase in strandings, as well as an increase in some marine mammal populations—especially pinnipeds—which may favor increased prevalence of density-dependent diseases [27]. An increased number of scientific researchers as well as enhanced investigations through interdisciplinary collaborations in some areas may have resulted in a subsequent increase in scientific effort on the study of marine mammal diseases [33]. These factors, along with multiple anthropogenic factors such as increasing recreational use of beaches and oceans and public awareness and concern for marine mammals and their environment likely explain the higher number of disease reports from California. However, they also prevent conclusions about increasing marine mammal disease from enhanced reporting alone from being drawn on a large scale.

Understanding disease trends is important, as health issues can highly influence marine mammal populations. Consistent monitoring and reporting is vital to accurately assess disease trends. Managers cannot expect to rely on publications alone to meet this need, but at the present time, these publications are the most comprehensive tool available to do so. This study highlights several issues with relying on publications to interpret disease trends. First, there is a discrepancy between the increasing trend in number of annual publications and the decrease in cumulative calculated cases after 2004. This may be partly due to a lag in publication following data collection. Furthermore, publications that reported pathogen isolation in the absence of disease corresponded with the emergence of novel diagnostic techniques to detect the pathogen rather than true emergence of a novel pathogen. For example, novel techniques to detect viruses, such as polymerase chain reaction (PCR), were not developed until the 1980s, resulting in a coincident increase in reporting on viral diseases at this time. Additionally, in many cases, the link between presence of a pathogen and evidence of clinical disease may not yet be achieved. As was specifically encountered in several reports of bacterial infection, the presence of a lesion and a pathogen does not immediately clarify whether the detected pathogen caused or contributed to the lesion or was unrelated (e.g., an incidental finding). Reporting of bacterial flora is important to more fully characterize the natural history of marine mammals, but increased focus on the impact of these organisms on host health is needed.

In this paper we referred to “calculated cases” rather than assuming actual annual number of cases were obtained from reports because there were multiple biases associated with determining the calculated cases. Multiple publications that may have reported the same cases were included in this study unless it was explicitly stated that there was overlap in case reporting. Thus, this dataset likely contained duplication of some cases reported in multiple publications. For example, calculated cases of biotoxicoses for California in 1998 resulted from nine publications reporting domoic acid toxicosis in California sea lions, but these reports overlapped spatially and temporally. Although we strongly suspect that many of these publications reported duplicates of the same individual cases, because the papers did not give sufficient details on case identification to indicate case overlap, all cases from these nine publications were included. Thus, results were likely biased by an over-reporting of cases.

In contrast, under-reporting of marine mammal diseases, injuries, and biotoxicosis also make it difficult to interpret marine mammal health trends. Cases were excluded from 17% of publications that lacked dates for data collection. For some publications that spanned a large time period, published figures may have showed changes in the number of cases over time, but the raw data could not be extracted from the graph, and cases for this review were instead equally distributed across all years for the study period. This will misrepresent short-term disease trends as well as dampen longer-term health trends. The calculated cases extracted from published reports cannot be used to determine true prevalence or incidence. Notably, most studies did not report the total number of animals that were assessed, but did not have the disease or pathogen of interest. This critical information is needed to determine disease prevalence. In addition, lack of standardization in terminology across taxa leads to inconsistently assigned cases. For instance, in sirenians, anthropogenic trauma is the most commonly reported cause of death. A complete dataset exists that assigns causes of death for every manatee case. Biotoxicosis and infectious disease cases, however, are grouped with congenital defects in a “natural causes, other” health category. Because these cases were not reported in the same manner as other marine mammal cases, and could not be separated into the categories used in this study, they were omitted from this study, creating a large gap in some species or geographic ranges.

There also are differences in the level of interest and funding various marine mammal diseases generate, which also leads to biases in reporting. Marine mammal diseases that cause significant morbidity or mortality such as morbillivirus or leptospirosis are fairly well represented in the literature, as large epizootics attract attention and concern, and often meet publication criteria, whereas reports of small disease outbreaks and endemic disease often do not meet publication standards. The literature provided little information on the pathogenicity of the agents involved in epizootics, and did not systematically differentiate between fatalities and cases of clinical disease with resolution and survival. In addition, zoonotic agents such as *Leptospira*, *Brucella*, and influenza carry marked public health significance, and surveillance may be increased for these pathogens, although their pathogenicity varies markedly among species.

Endemic diseases that sporadically affect smaller numbers of animals, such as fungal and protozoal infection, or produce widespread, low-level morbidity or a reduction in fitness or resilience, such as helminth infestation, often are only included in publications that retrospectively assess mortality trends in a certain species or region. This led to an elevation in calculated cases during the study period reported in the publication. For instance, a peak in the number of fungal calculated cases in 2003 resulted from a single publication that estimated prevalence of lacaziosis in a study population over two years [34]. This reflects a short period of reporting of endemic chronic skin lesions present in other years, rather than an outbreak of fungal cases in the southeast U.S. in those two years. Managers cannot rely on published literature to provide consistent information on prevalence of endemic disease over time because it is reports of novel discoveries that are of interest to the scientific community, and by extension to peer-reviewed journals. Publications can provide valuable baseline data for guiding future studies, and researchers should strive to share and include all available data—including such basic animal information as species and date of sample collection—to ensure that peer-reviewed publications are useful resources for future comparative studies.

A comparison between numbers of urogenital carcinoma in California sea lions at TMMC listed in the internal database and calculated cases of all reported neoplasia in California sea lions was used to highlight the issues of both over- and under-reporting. First, higher numbers of cases were reported than were found in the TMMC database in certain years, especially prior to 2000 (Fig 5). This may be due to cases diagnosed at other California facilities or with other types of neoplasia, but is most likely due to a duplication of reported cases due to individual animals being sampled for different studies such as genetics, toxicology and virology, and animal identification not reported in the published paper. Second, reported neoplasia cases ceased after 2003, while annual numbers of cases of urogenital carcinoma in the TMMC database increased after 2004 and peaked in 2012. This is likely due to the phenomenon that neoplasia cases are only likely to be reported when they are associated with a novel finding. These data thus suggest that cases of urogenital carcinoma have been increasing in California since 2004, but a novel aspect of the disease was last reported using cases from 2003 [35]. Similar biases were noted in other disease categories, and highlight the limitations of using published data to evaluate disease trends.

One goal of the MMPA was to reduce anthropogenic marine mammal mortalities in the U.S. This study clearly shows that the MMPA has not eradicated anthropogenic trauma in marine mammals. It is important to note that this study did not aim to summarize total marine mammal anthropogenic trauma cases, as fisheries by-catch is estimated to number more than 6,000 animals annually across the U.S., and in the hundreds of thousands globally [12]. Furthermore, under the 1994 Title IV amendment of the MMPA, the National Marine Fisheries Service (NMFS) was mandated to gather data on marine mammal health trends and correlate these with biological, physical and chemical variables [9]. The effect that disease, anthropogenic impact, and climate change are having on marine mammal health and the trajectory of their populations is unable to be assessed from a review of published literature. Currently, it is not possible to achieve either of these federal mandates using published literature, and no alternative accessible source of marine mammal disease data exists that can be overlaid with environmental data. This study highlights the difficulties in using peer-reviewed publications for assessing marine mammal health trends, due to lack of detail and inconsistencies in reporting and the strong influence of novel diagnostic techniques and scientific research priorities on publication rates. The efficacy of management and mitigation measures in marine mammal disease trends cannot be assessed using the current data sources.

At present, there is no centralized data reporting system for marine mammal health and disease data to allow detection of true trends in health and disease [36][17]. To overcome the weaknesses in marine mammal disease reporting described in this paper, we recommend the use of a database repository to provide access to the wealth of marine mammal data already being collected across the nation. This platform will: 1) standardize data reporting of marine mammal health findings across species and geographic regions; 2) integrate multiple data sources, including stranding response, entanglement response, live capture-release or research investigations, by-caught animals, and harvested animals; 3) provide a summary of health and disease trends to aid the public and resource managers in identifying potential epizootics or public health risks; and 4) enable rapid communication and analysis of health data in conjunction with environmental data and other sources of complementary disease data from terrestrial wildlife [36]. Much of the data discussed are already being collected by the data sources discussed above, yet data standards and mechanisms for sharing the data on a national scale are lacking. By enabling standardized tracking of whether a pathogen or disease state is present or absent, true disease prevalence reporting would be possible for the first time for marine mammal data. The Marine Mammal Health Monitoring and Analysis Platform (MMHMAP) is a proposed program to address these goals in the United States. The MMHMAP is currently in the development stage. In the absence of raw data, this paper provides a collated list of marine mammal disease publications for reference, designed to bridge historical knowledge until it can be entered into the MMHMAP (S1 Appendix). Significant resources and collaboration must be devoted to ensure success of this program.

Examples of successful, integrated marine mammal databases exist in Canada (through the Canadian Wildlife Health Cooperative) and in the United Kingdom (through the Cetacean Strandings Investigation Programme). In the United States, an integrated terrestrial wildlife database was recently started to track wildlife disease outbreaks, named the Wildlife Health Information Sharing Partnership (WHISPers, through the United States Geologic Survey). Collaboration is needed, not only across regional boundaries, but also across disciplines, agencies, institutions, and even international boundaries, to obtain an accurate measure of marine mammal health trends, and to learn from platforms already in use. As the ocean environment changes, it is vital to coordinate efforts and provide ready access to integrated marine mammal health data, so management and conservation efforts can be prioritized to improve marine mammal health, and marine mammal health data can be integrated into ocean health indices.

## Supporting Information

S1 Table PRISMA checklist for systematic review. (PDF)

S1 Appendix Publications included in qualitative synthesis of systematic review. Publications are sorted by health category, and may appear in more than one category if cases were characterized by more than one disease. Category details provided additional information about specific disease. A star is noted in the “omitted” column if study was omitted from quantitative synthesis due to lack of sampling date.
(XLSX)
